# Comparative efficacy of non-pharmacological interventions for post-stroke cognitive impairment: a systematic review and network meta-analysis of randomized controlled trials

**DOI:** 10.3389/fneur.2026.1644663

**Published:** 2026-03-03

**Authors:** Hongmei Niu, Weili Li, Lidong Du, Xin Zheng, Jin Liang, Xiujuan Yang, Jun Luo

**Affiliations:** 1 Northwest Minzu University, Gansu, China; 2 Key Laboratory of Environmental Ecology and Population Health in Northwest Ethnic Regions, State Ethnic Affairs Commission, Gansu, China; 3 Department of Neurology, The First Affiliated Hospital of Shandong First Medical University & Shandong Provincial Qianfoshan Hospital, Jinan, China; 4 Gansu University of Chinese Medicine, School of Pharmacy, Gansu, China; 5 Gansu Provincial People’s Hospital, Gansu, China; 6 Gansu University of Chinese Medicine, School of Integrated Chinese and Western Medicine, Gansu, China

**Keywords:** cognitive training, network meta-analysis, non-pharmacological interventions, post-stroke cognitive impairment, transcranial brain stimulation

## Abstract

**Background:**

Post-stroke cognitive impairment (PSCI) substantially diminishes quality of life and functional independence in stroke survivors. Various non-pharmacological interventions have been proposed to improve cognitive and functional outcomes; however, their relative effectiveness remains uncertain.

**Methods:**

A network meta-analysis of 23 RCTs (1,723 participants) evaluated seven non-drug therapies, including computer-based cognitive training (CCT), transcranial direct current stimulation (tDCS), repetitive transcranial magnetic stimulation (rTMS), acupuncture, exercise, and their combinations. Primary and secondary outcomes were MoCA and MBI scores, respectively.

**Results:**

Regarding MoCA scores, the most effective intervention was CCT combined with tDCS (mean difference vs. control: 6.67; 95% CrI: 1.20–12.13), followed by acupuncture combined with rTMS (6.59; 95% CrI: 4.34–8.84) and rTMS alone (4.26; 95% CrI: 2.65–5.88). SUCRA rankings indicated that CCT + tDCS and acupuncture + rTMS had the highest probabilities of being the most effective treatments. For MBI scores, tDCS (8.41; 95% CrI: 4.50–12.32), exercise rehabilitation (6.87; 95% CrI: 4.92–8.82), and CCT (6.62; 95% CrI: 3.84–9.39) demonstrated the greatest improvements compared to control. Funnel plots revealed no significant publication bias, and contribution plots supported the stability of the network geometry.

**Conclusion:**

Among non-pharmacological approaches for PSCI, combined CCT and tDCS produced the most consistent cognitive improvements, while tDCS and exercise rehabilitation yielded the most pronounced gains in functional recovery. These findings support the clinical integration of multimodal brain stimulation and cognitive rehabilitation strategies in the management of PSCI.

## Introduction

Stroke remains a leading cause of long-term disability worldwide, with over 16 million new cases reported annually (1). Globally, the prevalence of PSCI is alarmingly high, affecting approximately 20 to 80% of survivors depending on diagnostic criteria and geographic regions (2). Longitudinal studies across Europe and North America indicate that PSCI is a primary driver of post-stroke institutionalization and imposes a substantial long-term economic burden (3). Although pharmacological agents such as donepezil and memantine have been investigated, their clinical efficacy remains modest and is often constrained by adverse effects or potential resistance (4). Consequently, there is an urgent need for non-pharmacological interventions that offer safer, more sustainable avenues for post-stroke cognitive rehabilitation.

In recent years, several conventional meta-analyses have synthesized evidence for individual non-pharmacological modalities, such as CCT, exercise rehabilitation, and neuromodulation. For instance, previous reviews have confirmed the efficacy of rTMS and tDCS in modulating neural plasticity (5), while meta-analyses of CCT have demonstrated gains in executive function (6). Furthermore, traditional Chinese medicine techniques, including acupuncture and electro-acupuncture, have shown potential in enhancing neuro-recovery (7). However, most existing reviews have prioritized cognitive endpoints in isolation, without concurrent evaluation of functional independence—an equally critical component of stroke recovery.

More importantly, traditional pairwise meta-analyses are restricted by their methodology, as they typically compare a single active intervention against a control group (5). This leaves a critical gap in clinical knowledge: direct comparisons of relative efficacy across diverse modalities are lacking, and no comprehensive analysis has systematically ranked these approaches to inform evidence-based decision-making.

To address these gaps, we conducted a network meta-analysis (NMA) incorporating all eligible randomized controlled trials (RCTs) evaluating non-pharmacological interventions for PSCI. The novelty of this study lies in its application of an NMA framework, which allows for the simultaneous integration of direct and indirect evidence across a broad spectrum of therapies. By evaluating both the Montreal Cognitive Assessment (MoCA) and the Modified Barthel Index (MBI), this study aims to provide a hierarchical ranking of interventions, identifying which specific strategies or combinations (e.g., CCT combined with tDCS) yield the greatest clinical benefit. These findings are intended to guide individualized rehabilitation planning and promote the clinical adoption of the most effective non-drug therapies for PSCI management.

## Methods

### Literature search and study selection

A comprehensive literature search was conducted in five databases—PubMed, Embase, the Cochrane Library, Web of Science, and China National Knowledge Infrastructure (CNKI)—from inception to May 2025. The search strategy combined Medical Subject Headings (MeSH) and free-text terms, using Boolean operators to include terms such as “stroke,” “PSCI,” “cognitive dysfunction,” “non-pharmacological intervention,” “cognitive training,” “neuromodulation,” and “RCT (Supplementary File 1)”.

Studies were included if they met the following criteria: (1) RCT design; (2) inclusion of patients diagnosed with PSCI; (3) evaluation of non-pharmacological interventions, including cognitive training, brain stimulation, or physical rehabilitation; and (4) reporting of at least one of the following outcomes: the MoCA or the MBI. Exclusion criteria were: (1) studies without randomization; (2) interventions involving pharmacological agents; (3) duplicate publications; or (4) insufficient outcome data for analysis.

Two reviewers independently screened titles, abstracts, and full texts. Discrepancies were resolved by discussion with a third reviewer until consensus was reached.

### Definition of combination therapies

In this NMA, combination therapies were defined as interventions in which two distinct non-pharmacological modalities (e.g., CCT and tDCS) were administered to the same participant group within the same randomized treatment arm. To be classified as a combination therapy, studies were required to meet the following criteria: Both intervention components were delivered during the same treatment period, either concurrently (e.g., tDCS applied immediately before or during cognitive training sessions) or sequentially within each session or treatment week. Each component had a clearly defined protocol, including stimulation parameters (for tDCS or rTMS) and training content or intensity (for CCT or rehabilitation). The total intervention duration was at least 3 weeks, and the frequency and duration of each component were consistent across participants within the same study arm. The combined intervention was compared against a control group or a single-modality intervention within a RCT design. Studies in which different therapies were delivered to separate groups, or where interventions were applied in distinct, non-overlapping phases without integration, were not considered combination therapies and were analyzed as separate intervention nodes.

The Control group included participants receiving standard care or usual rehabilitation, sham interventions (sham tDCS, sham rTMS, or sham acupuncture), or no intervention. Standard care typically comprised routine post-stroke therapy without additional cognitive or neuromodulatory interventions. Sham procedures were designed to mimic the active intervention without delivering therapeutic stimulation. Combining these conditions into a single Control node allowed consistent indirect comparisons across interventions while maintaining network connectivity.

### Data extraction and outcome measures

Two reviewers independently extracted data using a standardized form. Extracted variables included first author, year of publication, country, sample size, mean participant age, sex distribution, intervention and control conditions, treatment duration, and outcome measures.

The primary outcome was cognitive function, assessed by MoCA. The secondary outcome was functional ability, assessed using MBI.

### Risk of bias assessment

The methodological quality of each included study was evaluated using the Cochrane Risk of Bias 2.0 tool (8). This tool assesses five domains: (1) random sequence generation; (2) allocation concealment; (3) blinding of participants, personnel, and outcome assessors; (4) completeness of outcome data; and (5) selective outcome reporting. Each study was rated as having low risk of bias, some concerns, or high risk. Risk-of-bias summaries were visualized using RevMan (version 5.4).

The overall certainty of evidence for each primary outcome was evaluated using the Grading of Recommendations Assessment, Development and Evaluation (GRADE) approach. Evidence derived from RCTs was initially rated as high certainty and subsequently downgraded based on five domains: risk of bias, inconsistency, indirectness, imprecision, and publication bias. For NMA outcomes, the GRADE assessment focused on the overall body of direct and indirect evidence contributing to each outcome. The final certainty of evidence was classified as high, moderate, low, or very low. A detailed summary of findings and GRADE assessments is provided in the Supplementary Online Content (Supplementary Table 3).

The confidence in the evidence for each pairwise and network comparison was assessed using the Confidence in Network Meta-Analysis (CINeMA) framework. CINeMA evaluates six key domains: within-study bias, reporting bias, indirectness, imprecision, heterogeneity, and incoherence. Within-study bias was calculated based on the contribution of each study’s risk of bias (Cochrane RoB 2.0) to the network estimates. Incoherence was assessed by the disagreement between direct and indirect evidence. The final confidence ratings were categorized as High, Moderate, Low, or Very Low.

### Statistical analysis

A Bayesian NMA was conducted using Markov Chain Monte Carlo (MCMC) simulation to estimate the relative effectiveness of the included interventions. A random-effects model was employed to account for between-study heterogeneity. Effect sizes for continuous outcomes were reported as mean differences (MDs) with 95% credible intervals (CrIs). To rank the treatments, surface under the cumulative ranking curve (SUCRA) values were calculated, where higher SUCRA scores indicate a higher probability of being among the most effective interventions. All statistical analyses were performed using STATA software.

To investigate potential sources of heterogeneity related to intervention protocols, pre-specified subgroup analyses were conducted focusing on key treatment parameters of tDCS. Specifically, studies were stratified according to treatment duration (4 weeks vs. 8 weeks) and stimulation intensity (1, 1.3, and 2 mA). Subgroup analyses were performed for cognitive outcomes measured by the MoCA.

### Assessment of network inconsistency

To evaluate the consistency between direct and indirect evidence within the network, we performed a formal inconsistency assessment using the node-splitting approach. This method separates evidence for a given comparison into direct and indirect components and statistically tests their agreement. Node-splitting analyses were conducted for all eligible comparisons within the NMA. A *p*-value greater than 0.05 was considered indicative of no significant inconsistency. Detailed results of the node-splitting analyses for both MoCA and MBI outcomes are provided in the Supplementary Online Content.

### Sensitivity analysis and publication bias

Sensitivity analyses were conducted by sequentially removing individual studies from the network to test the robustness of the findings. Potential small-study effects and publication bias were assessed using comparison-adjusted funnel plots. To evaluate the structural integrity of the network, contribution plots and network geometry diagrams were generated.

## Results

### Study selection

**Figure 1:**
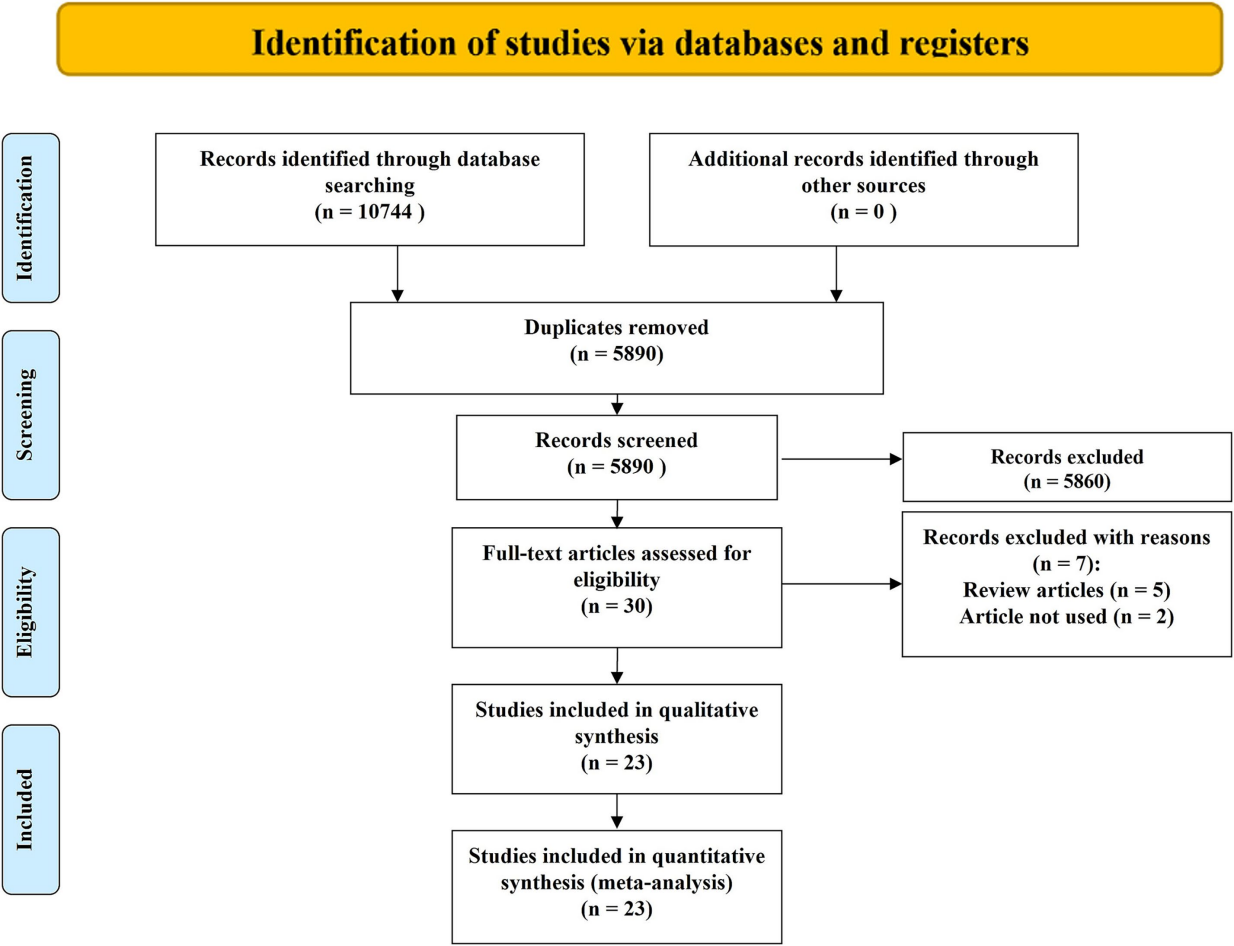
Study identification and selection.

A total of 10,744 records were identified through database searches. After removing 5,860 duplicates, 4,884 records remained for title and abstract screening. Of these, 5,830 were excluded due to irrelevance, and 30 full-text articles were assessed for eligibility. Ultimately, 23 RCTs comprising 1,723 participants were included in the NMA (9–30). The study selection process is presented in Figure 1.

### Study characteristics

The included RCTs were published between 2019 and 2025. Most studies were conducted in China (*n* = 21), with one study each from Italy, Korea, and the United States. Sample sizes ranged from 26 to 132 participants. All studies enrolled individuals diagnosed with PSCI and evaluated the efficacy of various non-pharmacological interventions.

The intervention strategies included computerized cognitive training (CCT; 11 studies); tDCS, either alone or in combination with other therapies (5 studies); rTMS or intermittent theta burst stimulation (iTBS; 4 studies); electro-acupuncture or scalp acupuncture-based interventions (5 studies); exercise rehabilitation programs (2 studies); and combination therapies such as CCT plus tDCS or acupuncture combined with rTMS (4 studies).

Treatment durations ranged from 3 to 24 weeks, with most studies administering interventions for 4–8 weeks. The MoCA was used as the primary outcome measure of cognitive function in 22 studies, while the MBI was used to assess functional ability in 6 studies. Additional instruments, including the Mini-Mental State Examination (MMSE) and ADL scales, were reported in a minority of trials.

Participant demographics were generally comparable across studies, with mean ages ranging from the mid-50s to late 60s and a relatively balanced sex distribution. Participants were enrolled at different stages of stroke recovery, ranging from the subacute to chronic phase. The severity of cognitive impairment varied across trials, encompassing mild, moderate, and, in a few studies, severe impairment. Common baseline comorbidities included hypertension, diabetes mellitus, hyperlipidemia, and other cardiovascular conditions.

To enhance transparency and facilitate a more comprehensive evaluation of clinical heterogeneity and intervention comparability, detailed information regarding participant baseline characteristics and intervention protocols—including stimulation targets, frequencies, intensities, session durations, and total treatment periods—is summarized in Table 1, while a general overview of study characteristics is presented in Table 2.

**Table 1:** Detailed intervention protocols and participant baseline characteristics of included studies.

Author (year)	Baseline characteristics			Intervention protocols		Stimulation parameters		
	Time since stroke	PSCI severity	Baseline comorbidities	Type	Detailed Parameters	Frequency	Intensity	Duration
Chen et al. (2020) (9)	Within 3 to 6 months of stroke onset.	Mild to moderate cognitive impairment.	Cerebrovascular diseases (subcortical ischemic infarction or cerebral hemorrhage); excluded patients with severe heart, liver, or kidney diseases.	Combination therapy: scalp acupuncture with cluster needling plus drug treatment (donepezil).	Seven scalp regions (e.g., parietal, frontal, temporal); needles (0.25 mm * 40 mm) inserted 0.5–1 inch deep.	Twice a day for 6 days per course; total of 4 treatment courses.	Strong stimulation (200 rpm) to create a “needle field” and increase cortical excitability.	1 month of consecutive treatment (acupuncture courses plus daily drug dose).
Zheng et al. (2020) (10)	Mean 6.58 ± 2.14 months.	Baseline MoCA scores ranged from 20.71 ± 0.51 to 21.21 ± 0.84 (indicating mild cognitive impairment), with an average NIHSS score of 2.77 ± 1.17.	Includes hypertension (45 participants), hyperlipidemia (38 participants), and hypoglycemia (25 participants).	Baduanjin exercise based on routine medical and rehabilitation treatment.	Group training at community centers, guided by qualified coaches.	3 days a week	Mild to moderate intensity	40 min a day for 24 weeks.
Manuli et al. (2020) (11)	Chronic phase, at least 6 months from the acute event. (mean interval: 4.7 ± 1.3 months).	Presence of mild to moderate cognitive impairment (MoCA from 16 to 24).	Neurological diagnosis of stroke (ischemic or hemorrhagic). Exclusion of disabling sensory alterations (hallucinations) and concomitant medical/psychiatric illnesses.	RRG + VR: robotic rehabilitation plus virtual reality group using Lokomat-Pro with a VR screen. RRG-VR: robotic rehabilitation group without VR using Lokomat Nanos. CRG: conventional rehabilitation group submitted to conventional physical and cognitive therapy.	Body weight support: initially set at 70% of the patient’s weight, reduced based on tolerance to no less than 20%. Speed: selected speed adapted to the patient’s need under the supervision of a physiotherapist. VR feedback: interactive exercises (collecting/avoiding objects) projected on a flat screen, providing personalized feedback connected to the patient’s walk.	5 times a week.	Each session lasted about 1 h. (All groups received equal total rehabilitation time).	8 weeks (total of 40 sessions).
Youze et al. (2021) (12)	Patients were within one year poststroke.	Cognitive impairment was identified by MoCA scores between 10 and 26 points.	Patients with a history of mental retardation, dementia, severe mental disorders, or serious systemic diseases (heart, liver, or renal failure; malignant tumors; gastrointestinal bleeding) were excluded.	A combination of Computer-Aided Self-Regulation Learning (CA-SRL) and Computer-Aided Cognitive Training (CACT).	CA-SRL: Includes step decomposition, self-calibration (using video reflection), and mental imagery. CACT: Includes a basic cognitive module (reaction time, perception, attention, memory) and a cognitive application module.	5 sessions per week.	90 min per day total: 1 h for SRL/ADL training and 30 min for CACT	3 weeks total.
Liu et al. (2021) (13)	6–18 months (Chronic stage).	MMSE score ≦24 and MoCA score ≦26; including mild, moderate, and severe cognitive impairment.	Stable condition, excluding severe cardiopulmonary, renal, hepatic diseases, or psychiatric disorders.	Combined anodal tDCS and computer-assisted cognitive training.	Computer-assisted training: structural ability, reasoning ability, and simulated daily task-oriented training. Target: Anode over the left DLPFC, cathode over the right DLPFC.	5 sessions per week for 4 weeks (Total 20 sessions)	2.0 mA	4 weeks total.
Ko et al. (2022) (14)	Chronic stage (≥6 months after onset)	Korean version of the Montreal cognitive assessment (K-MoCA) score <26.	Including ischemic and hemorrhagic stroke; excluding severe neuropsychiatric disorders, history of epilepsy, metallic intracranial implants, or severe cognitive impairment (K-MoCA < 9)	Remotely supervised transcranial direct current stimulation (RS-tDCS) concurrent with computerized cognitive training (CogTx)	Anode over left DLPFC (F3) and cathode on the right supraorbital area, 28.3 cm^2^ * 28.3 cm^2^, memory and attention-based tasks (Comcog program)	5 sessions per week for 4 weeks (total 20 sessions)	2.0 mA	4 weeks total.
Huang et al. (2021) (15)	NR.	Participants had vascular cognitive impairment with no dementia (VCIND), evidenced by baseline MoCA scores below 26.	Common comorbidities among participants included hypertension, coronary heart disease, diabetes mellitus, and hyperlipidemia.	The intervention was a randomized comparison between electro-acupuncture (EA) and non-invasive Sham acupuncture (SA).	Treatment involved specific acupoints including GV20, GV29, GV24, GV26, GV17, EX-HN1, and bilateral GB20, HT7, and SP6. Electro-acupuncture was applied to GV20 and GV29 using a G6805-2 device at a frequency of 2.5 Hz.	The therapeutic regimen consisted of 24 sessions administered thrice weekly over a period of 8 weeks.	Current intensity was set between 2-3 mA based on patient comfort after achieving the “Deqi” sensation through manual manipulation.	8 weeks total.
He et al. (2021) (16)	Disease course ≤6 months.	MoCA <26 points.	Excluded: severe heart/liver/kidney dysfunction, respiratory failure, tumors, mental illness, drug/alcohol dependence.	Cognitive rehabilitation strategy based on eye movement technique.	Routine rehab (PT/OT/ST) + 20 min eye-move training (goal management, working memory, and inhibitory control), eye movement visual training (5 min): track ball, skiing (tracking), fruit cutting (gaze), commodity selection, electronic organ training	Once a day, 6 days a week	Total training duration of 80 min per day	6 weeks of intervention, followed by a 4 week follow-up
Wang et al. (2021) (21)	Time since stroke: observation group: 35.54 ± 10.55 d; control group: 32.24 ± 9.92.	PSCI severity: MoCA score ≤24 points. Baseline MoCA total: observation group:17.03 ± 2.56; control group: 16.05 ± 2.26.	Includes hypertension, diabetes, and hyperlipidemia.	Anodic transcranial direct current stimulation (tDCS) combined with donepezil.	Donepezil hydrochloride tablets (5 mg), once daily, taken before bedtime for 4 weeks. Anode placed over the affected dorsolateral prefrontal cortex (DLPFC); cathode placed over the contralateral supraorbital region.	Once daily, 6 times per week.	2.0 mA	4 weeks total.
Cheng et al. (2024) (22)	More than 1 month.	Cognitive impairment caused by stroke. Baseline MoCA scores: CCT group (17.86 ± 2.07); tDCS combined group (18.21 ± 2.11).	Excluded severe cognitive impairment before onset, brain surgery, epilepsy, brain trauma, psychiatric diseases, and severe organ dysfunction.	tDCS combined with CCT.	Six-Six brain rehabilitation system: memory, attention, perception, agility, executive ability, and calculation/reasoning training. IS300 tDCS device: anode at the ipsilateral dorsolateral prefrontal cortex (DLPFC), cathode at the contralateral shoulder.	Once daily, 5 times per week, for 8 weeks.	1.0 mA	8 weeks total.
Meng et al. (2022) (23)	Mean course of disease approximately 8–9 weeks (subacute stage).	Mild-to-moderate cognitive impairment: inclusion criteria MMSE <24; baseline total scores were 18.38 ± 3.25 (Exp) and 17.61 ± 2.56 (Con).	Excluded severe medical diseases, Alzheimer’s disease, and history of alcoholism or drug abuse.	Computer-assisted cognitive rehabilitation (CACR)	Combined multi-domain occupational therapy (upper limb control, fine motor skills, and cognitive tasks like memory and language) with DK-YYZ system-based CACR focusing on digitized attention, memory, spatial logic, and calculation training.	Once daily, 5 times per week.	30 min/session	3 months
Yao et al. (2021) (24)	Within 6 months post-stroke; control group: (5.9 ± 1.5) months; treatment group: (6.2 ± 0.9) months 4.	Mild cognitive impairment.	Exclusion of severe visual/auditory/speech/mental disorders, malignant tumors, severe chronic medical diseases, or other intracranial diseases.	Computer-assisted cognitive intervention training (CACIT) combined with donepezil and secondary prevention	Five functional modules: attention, memory, calculation, reasoning/thinking, and perception training, interactive cognitive tasks via a computerized system (specific software-based intervention).	30 min per session, 5 sessions per week	Intensive cognitive training (30 min/session, once daily, 5 days/week)	6 weeks total.
Luo et al. (2020) (25)	NR.	Mild to moderate cognitive impairment (MoCA score 10–26).	Hypertension, diabetes mellitus, hyperlipidemia, etc.	Computer-assisted attention rehabilitation system (CAARS).	Includes attention training (visual tracking, go-no-go tasks) and comprehensive cognitive rehabilitation. Interactive visual and auditory feedback (based on computer programs).	5 days per week.	60 min per session.	12 weeks total.
Ling and Ran (2020) (43)	NR.	Baseline MoCA score approx. 16 (indicating presence of cognitive impairment)	Cerebral infarction (*n* = 30), cerebral hemorrhage (*n* = 30)	Computer-aided cognitive intervention training (CACIT) combined with conventional medication and rehabilitation	Phased training: including attention, observation, numerical cognition, memory, graphic recognition, sequence distribution, classification, advanced reasoning, and situational calculus	NR.	Step-wise intensity: progressing from basic cognitive tasks to advanced reasoning and memory strategies	NR.
Xin et al. (2023) (30)	Average course of disease: western medicine group (2.71 ± 1.04) months; acupuncture-rehabilitation group (2.84 ± 1.11) months.	Cognitive impairment after cerebral infarction; baseline MMSE: (17.86 ± 2.43) to (17.95 ± 2.64); baseline MoCA: (16.83 ± 2.69) to (16.74 ± 2.75).	Hypertension (40 cases), diabetes (46 cases), hyperlipidemia (22 cases).	Acupuncture-rehabilitation method combined with rTMS.	Acupuncture: “Scalp acupuncture-head-top cluster needle” + rehabilitation: 45 min/session + rTMS. target: left dorsolateral prefrontal cortex (DLPFC/F3); coil: “8” shape coil (70 mm).	20 min/session, 1 session/day, 5 days/week	5.0 Hz.	4 weeks (total 6,000 pulses/session).
Han et al. (2024) (26)	Control group: 4.32 ± 0.82 months; observation group: 4.47 ± 0.91 months.	Mild cognitive impairment (MCI)	Ischemic stroke patients with “stasis obstructing brain collaterals” type dementia	Electroacupuncture (EA) + rTMS + conventional treatment	Acupoints: Baihui (GV20), Shenting (GV24), Sishencong (EX-HN1), Fengchi (GB20), Yintang (GV29), Quchi (LI11). EA device: RHDC-A low-frequency electromagnetic pulse, target: left dorsolateral prefrontal cortex (DLPFC)	20–30 min/session, 1 session/day, 5 days/week, for 1 month	5.0 Hz.	4 weeks total.
Mao et al. (2020) (17)	3–9 months (subacute to early chronic stage)	Mild to moderate cognitive impairment (MoCA score ≥15)	Hemiplegia; excluded severe hypertension, cardiopulmonary disease, or mental disorders	Mirror neuron system-based training (MNST) combined with conventional rehabilitation	Action observation training (AOT) via VR glasses; watching 40 hand motion videos (e.g., daily tasks like cutting fruit) and imitating simultaneously	20 min/day, 5 days/week	40 daily hand action videos, each played 3 times per session	8 consecutive weeks
Koch et al. (2020) (18)	Stroke within 1 year.	MoCA mean score 19.5 (SD 5.6) for intervention and 20.7 (SD 5.7) for control.	Hypertension: 84% (intervention); 80% (control). Diabetes: 27% (intervention); 44% (control). Dyslipidemia: 70% (intervention); 67% (control)	Combined aerobic, resistance, and cognitive training (CARET + CTI)	Aerobic: stationary treadmill or bicycle ergometer. Resistance: 10 exercises on stacked-weight machines (e.g., leg press, chest press) plus core exercises. Cognitive: computerized platform targeting attention, memory, processing speed, and executive function.	3 sessions per week for 12 weeks.	Aerobic: moderate (up to 65% HRmax). Resistance: 1–3 sets of 8–15 repetitions. Cognitive: four 10 min training tasks per session	12 weeks total.
Chen et al. (2022) (27)	Observation group: (37.18 ± 10.52) days, Control group: (36.74 ± 10.23) days.	Baseline MMSE: 10.32 ± 2.01 (Obs) vs. 10.47 ± 2.16 (Con), baseline MoCA: 11.27 ± 2.11 (Obs) vs. 11.59 ± 2.46 (Con)	Exclusion: Severe TBI, brain tumor, organ dysfunction (liver/kidney), aphasia, visual/hearing impairment	tDCS combined with cognitive rehabilitation training	Cognitive training: attention, writing, memory, identification, and orientation training, anode: C3/C4 (M1 area), current mode: constant direct current stimulation cathode: bilateral orbits.	Once daily, 5 days per week, for a total of 28 days	1.3 mA	4 weeks total.
Wang et al. (2021) (29)	NR.	Mild to moderate cognitive impairment: MoCA score <26, MMSE score >17 (illiterate), >20 (primary school), or >24 (junior high and above).	Hemorrhagic stroke (intracerebral hemorrhage volume <30 mL).	High-frequency rTMS vs. Sham rTMS.	Target: left dorsolateral prefrontal cortex (DLPFC). Equipment: YS600 magnetic stimulator.	Once daily, 5 days per week.	5.0 Hz.	4 weeks total.
Gao and Qi (2019) (28)	NR.	Mild to moderate cognitive impairment; MMSE scores between 8 and 24	Hypertension, diabetes, and hyperlipidemia.	Study group: eye acupuncture combined with cognitive training; control group: routine cognitive training alone.	Eye acupuncture: specific zones including upper Jiao, lower Jiao, liver, and kidney. Cognitive training: memory, calculation, orientation, and language training.	Once daily, 5 days per week.	Manual acupuncture with needle retention for 30 min; cognitive training for 30 min per session.	8 weeks total.
Ren et al. (2025) (19)	Mean (SD): high-dose: 77.36 (32.70) days; standard: 83.15 (37.53) days; Sham: 70.93 (37.21) days (range: 1–12 months post-stroke)	MoCA baseline mean (SD): high-dose: 15.86 (7.04); standard: 14.15 (6.87); Sham: 18.07 (6.65).	First-time stroke, supratentorial lesion, stable vital signs, no severe pre-stroke cognitive impairment	iTBS targeting personalized frontoparietal cognitive network (FCN) + cognitive training	Personalized targets identified via 24 min resting-state fMRI; 15 workdays (3 weeks); daily CCT (30 min)	2 sessions/day, 200 s/session (1,200 pulses/day).	5.0 Hz.	4 weeks total.
Boyang et al. (2024) (20)	3–12 months.	Patients with mild cerebral infarction or cognitive impairment.	Stroke-related sleep disorders (SSD) and PSCI.	Electroacupuncture (EA) at Sishencong (EX-HN1) plus routine rehabilitation.	Acupoints: Sishencong (EX_HN1). Needle: 0.30 mm times 40 mm Depth: ~1.5 cm. Angle: <30° (toward the back of the brain).	20 min/session, 5 days/week	2 Hz continuous wave, adjusted to individual tolerance (approx. 2-3 W)	3 weeks total.

ADL, Activities of daily living; AOT, Action observation training; CACR, Computer-assisted cognitive rehabilitation; CACT, Computer-aided cognitive training; CAARS, Computer-assisted attention rehabilitation system; CACIT, Computer-aided cognitive intervention training; CA-SRL, Computer-aided self-regulation learning; CCT, Computerized cognitive training; DLPFC, Dorsolateral prefrontal cortex; EA, Electroacupuncture; fMRI, Functional magnetic resonance imaging; HRmax, Maximum heart rate; iTBS, Intermittent theta burst stimulation; MMSE, Mini-mental state examination; MoCA, Montreal cognitive assessment; NIHSS, National Institutes of Health Stroke Scale; NR, Not reported; OT, Occupational therapy; PSCI, Post-stroke cognitive impairment; PT, Physical therapy; rTMS, Repetitive transcranial magnetic stimulation; SA, Sham acupuncture; SSD, Stroke-related sleep disorders; ST, Speech therapy; tDCS, Transcranial direct current stimulation; VCIND, Vascular cognitive impairment no dementia; VR, Virtual reality.

**Table 2:** Characteristics of all studies and included arms.

Author (year)	Study design, location	Sample of study (*n*)		Age (years)		Sex (male/female)		Intervention measures		Treatment duration (weeks)	Assessment tool
		Intervention	Control	Intervention	Control	Intervention	Control	Intervention	Control		
Chen et al. (2020) (9)	RCT, China	28	28	61.00 ± 8.00	59.00 ± 9.00	17/11	16/12	Cluster needling scalp acupuncture on top of the drug treatment	Control: Drug treatment	4	MoCA, ADL
Zheng et al. (2020) (10)	RCT, China	24	24	61.63 ± 9.21	62.75 ± 6.41	17/7	19/5	Exercise rehabilitation: Baduanjin	Control: maintained original medication and rehabilitation treatment	24	MoCA, MBI
Manuli et al. (2020) (11)	RCT, Italy	30	30	48.00 ± 12.10	43.10 ± 9.70	19/11	16/14	CCT	Control: conventional rehabilitation	8	MoCA
Youze et al. (2021) (12)	RCT, China	23	25	57 (51, 65)	58 (51.5, 66)	19/6	19/6	CCT	Control: traditional cognitive training	6	MoCA, MBI
Liu et al. (2021) (13)	RCT, China	25	25	65 (60.5, 70.5)	64 (55.0, 70.0)	15/10	12/13	tDCS and cognitive training	Control: Sham tDCS and cognitive training	4	MoCA, ADL
Ko et al. (2022) (14)	RCT, Korea	12	14	61.25 ± 12.85	57.86 ± 10.04	4/8	8/6	tDCS and cognitive training	Control: Sham tDCS and cognitive training	4	MoCA
Huang et al. (2021) (15)	RCT, China	60	60	65.1 ± 7.5	64.6 ± 8.4	33/27	29/31	Electro-acupuncture	Control: Sham acupuncture	8	MoCA, MBI
He et al. (2021) (16)	RCT, China	32	32	66.0 ± 6.9	67.0 ± 5.9	17/15	18/14	CCT	Control: conventional rehabilitation	6	MoCA, MBI
Wang et al. (2021) (21)	RCT, China	39	41	59.64 ± 9.03	57.12 ± 9.26	25/14	26/15	tDCS and cognitive training	Control: conventional rehabilitation	4	MoCA, MBI
Cheng et al. (2024) (22)	RCT, China	66	66	56.30 ± 6.41	59.73 ± 4.62	40/26	38/28	CCT and tDCS	CCT	8	MoCA
Meng et al. (2022) (23)	RCT, China	40	40	56.25 ± 5.38	57.40 ± 6.04	23/17	26/14	CCT	Control: conventional rehabilitation	12	MMSE, MBI
Yao et al. (2021) (24)	RCT, China	40	40	68.6 ± 6.2	67.9 ± 6.5	23/17	25/15	CCT	Control: conventional rehabilitation	6	MoCA
Luo et al. (2020) (25)	RCT, China	30	30	56.42 ± 6.43	56.36 ± 6.45	15/15	13/17	CCT	Control: conventional rehabilitation	9	MoCA
Ling and Ran (2020) (43)	RCT, China	30	30	59.62 ± 0.85	60.72 ± 0.88	15/15	15/15	CCT	Control: conventional rehabilitation	6	MoCA
Xin et al. (2023) (30)	RCT, China	45	44	64.05 ± 5.17	62.34 ± 4.58	26/19	24/20	Needling scalp acupuncture and repeat transcranial magnetic stimulation	Control: repeat transcranial magnetic stimulation	4	MoCA
Han et al. (2024) (26)	RCT, China	53	53	65.33 ± 3.51	65.17 ± 3.61	30/23	29/24	Needling scalp acupuncture and repeat transcranial magnetic stimulation	Control: repeat transcranial magnetic stimulation	4	MoCA
Mao et al. (2020) (17)	RCT, China	30	30	54 ± 7	57 ± 6	16/14	15/15	CCT	Control: conventional rehabilitation	8	MoCA, MBI
Koch et al. (2020) (18)	RCT, USA	86	45	59 ± 11	58 ± 12	60/26	21/24	Exercise rehabilitation: combined aerobic, resistance, and cognitive training intervention	Control: maintained original medication and rehabilitation treatment	12	MoCA
Chen et al. (2022) (27)	RCT, China	36	36	64.01 ± 5.71	63.58 ± 5.48	18/18	19/17	tDCS and cognitive training	Control: Sham tDCS and cognitive training	4	MoCA
Wang et al. (2021) (29)	RCT, China	60	60	64.91 ± 4.15	65.3 ± 4.81	28/32	36/24	Repeat transcranial magnetic stimulation	Control: conventional rehabilitation	4	MoCA
Gao and Qi (2019) (28)	RCT, China	43	43	64.16 ± 7.42	65.81 ± 9.94	22/21	24/19	Electro-acupuncture and cognitive training	Control: traditional cognitive training	8	MoCA
Ren et al. (2025) (19)	RCT, China	14	14	58.57 ± 7.89	56.71 ± 9.96	11/3	11/3	rTMS (iTBS)	Control: Sham rTMS	4	MoCA
Boyang et al. (2024) (20)	RCT, China	34	33	63 ± 8	61 ± 7	16/18	15/18	Electro-acupuncture	Control: Sham acupuncture	3	MoCA

### Network structure and risk of bias assessment

**Figure 2:**
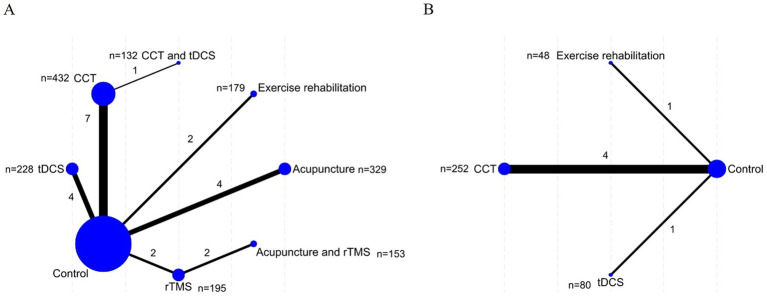
Network. **(A)** MoCA; **(B)** MBI. Each node represents an intervention, with node size proportional to the number of participants receiving that intervention. Edges denote direct comparisons between interventions, with line thickness proportional to the number of contributing studies. For reference, a line of minimum thickness corresponds to 1 study, and maximum thickness corresponds to 5 studies (or the maximum in this network).

The network structures for the MoCA and MBI outcomes are presented in Figure 2. In each network, nodes represent individual interventions, with node size proportional to the total number of participants allocated to each treatment. Edges indicate direct head-to-head comparisons, and edge thickness reflects the number of studies contributing to each comparison. Overall, the networks were well connected, allowing for the integration of both direct and indirect evidence across most interventions.

Risk of bias was evaluated using the Cochrane Risk of Bias 2.0 tool, with detailed assessments summarized in Supplementary Figures 1a,b. Most included studies were judged to have a low risk of bias in domains related to the randomization process and outcome measurement. However, some concerns were identified regarding allocation concealment and blinding, particularly in trials involving physical or acupuncture-based interventions, where blinding of participants and personnel was inherently challenging.

The certainty of evidence for each primary outcome was assessed using the GRADE framework, with results summarized in Supplementary Table 3. For cognitive outcomes assessed by the MoCA, the overall certainty of evidence was rated as low, primarily due to substantial heterogeneity among studies and concerns related to risk of bias. In contrast, for functional outcomes assessed by the MBI, the certainty of evidence was rated as moderate, reflecting highly consistent results despite the relatively limited number of included trials.

Confidence in the network estimates, as assessed by CINeMA, varied across comparisons (Supplementary Figure 10). For the MoCA outcome, several comparisons involving combination therapies were rated as ‘Low’ confidence, with Incoherence and Within-study bias identified as the primary reasons for downgrading. Conversely, for the MBI outcome, confidence ratings remained predominantly ‘Moderate’, reflecting higher consistency in functional recovery data despite the smaller number of trials.

### Network inconsistency assessment

Network inconsistency was assessed using the node-splitting approach. For both the MoCA and MBI outcomes, no statistically significant differences were observed between direct and indirect estimates across all evaluated comparisons (all *p-*values >0.05), indicating good agreement within the evidence network. The detailed node-splitting results are presented in the Supplementary Online Content.

### Comparative effectiveness and treatment rankings

**Figure 3:**
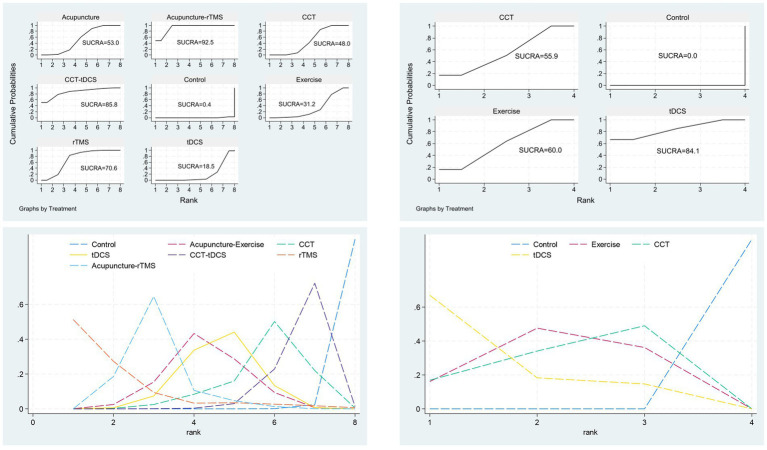
SUCRA plots for MoCA and modified barthel index.

**Figure 4:**
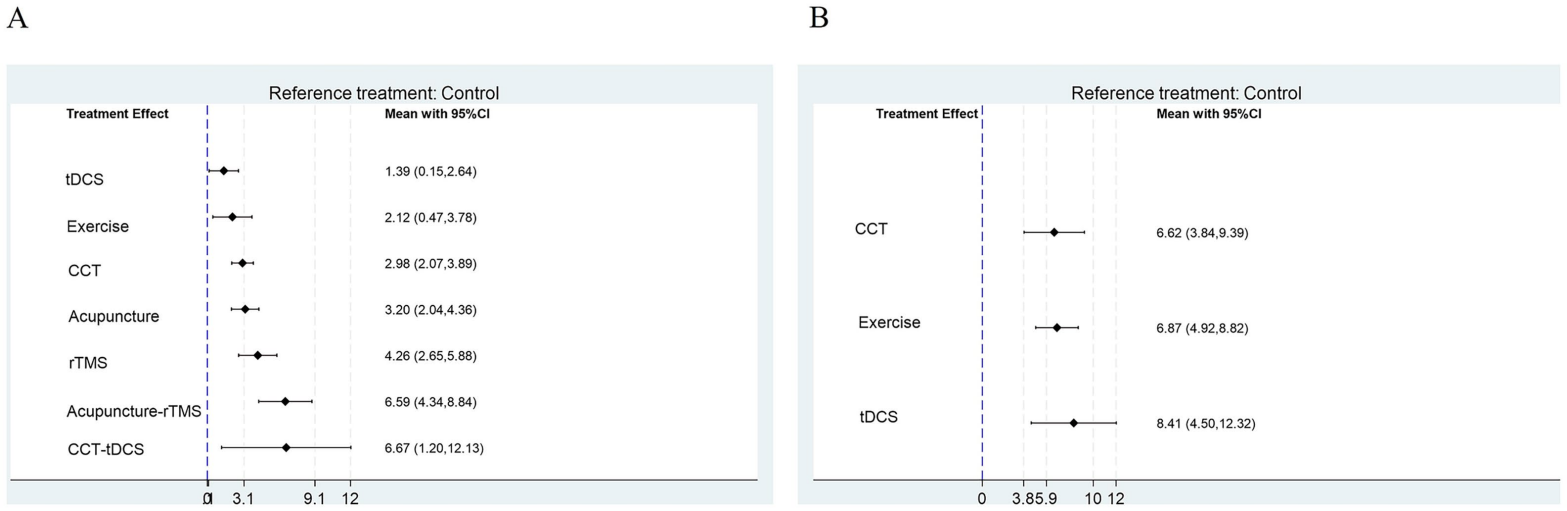
Forest plot for **(A)** MoCA and **(B)** MBI.

**Figure 5:**
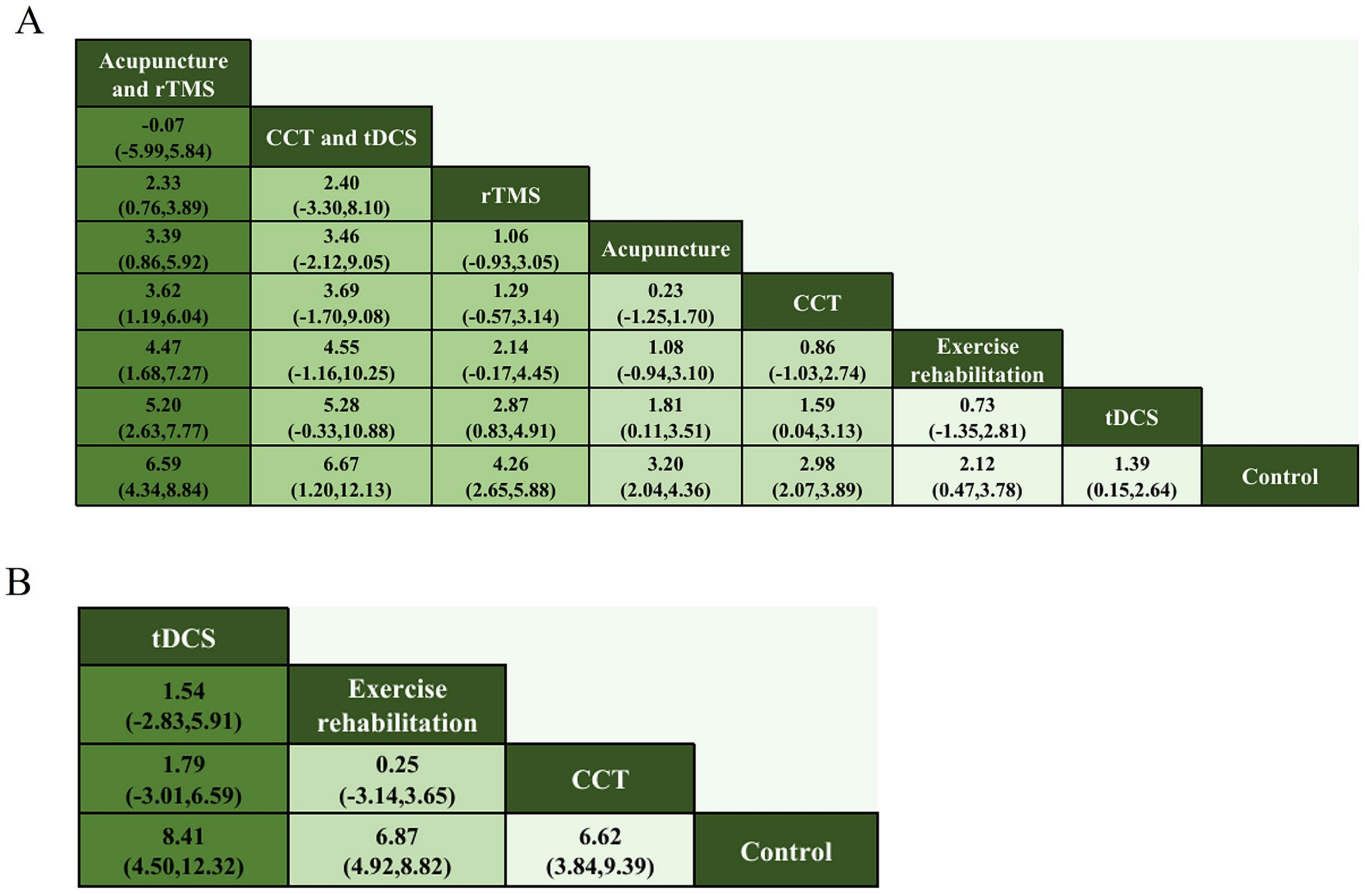
Network league of outcomes **(A)** MoCA; **(B)** MBI.

Comparative effectiveness estimates and treatment rankings are summarized in Figures 3–5. Figure 3 presents the cluster analysis based on SUCRA values for the two outcomes. The scatter plot facilitates a visual synthesis of treatment hierarchies: interventions such as CCT + tDCS and Acupuncture + rTMS were positioned toward the upper-right, indicating high probability of being the most effective treatments for both cognitive (MoCA) and functional (MBI) improvements. For cognitive outcomes assessed by MoCA, the most effective intervention was CCT combined with tDCS (MD vs. control: 6.67; 95% CrI: 1.20 to 12.13), followed by acupuncture combined with rTMS (MD: 6.59; 95% CrI: 4.34 to 8.84), and rTMS alone (MD: 4.26; 95% CrI: 2.65 to 5.88). For functional outcomes measured by MBI, tDCS alone produced the greatest improvement (MD: 8.41; 95% CrI: 4.50 to 12.32), followed by exercise rehabilitation (MD: 6.87; 95% CrI: 4.92 to 8.82) and CCT (MD: 6.62; 95% CrI: 3.84 to 9.39). SUCRA analysis confirmed that CCT + tDCS and acupuncture + rTMS ranked highest in terms of cognitive benefits, whereas tDCS and exercise rehabilitation were most effective for improving functional independence.

Clinical significance analysis confirmed that the primary cognitive gains achieved by CCT + tDCS and Acupuncture + rTMS were substantially higher than the MoCA MCID threshold of 1-2 points (31). Similarly, for functional independence (MBI), all major modalities demonstrated improvements that far surpassed the MCID of 1.85 points (32), with tDCS showing the most robust clinical impact (MD: 8.41).

### Assessment of heterogeneity

We evaluated statistical heterogeneity within each intervention node using *I*^2^ statistics for all pairwise comparisons contributing to the network. Statistical heterogeneity within intervention nodes was assessed using pairwise meta-analyses, and *I*^2^ statistics were calculated for each intervention contributing to the network. Detailed results are summarized in Supplementary Tables 1, 2. For MoCA outcomes, substantial heterogeneity was observed in several intervention nodes, including acupuncture (*I*^2^ = 85.8%), CCT (*I*^2^ = 70.3%), and exercise rehabilitation (*I*^2^ = 68.6%). In contrast, negligible heterogeneity was found for tDCS (*I*^2^ = 0.0%) and rTMS (*I*^2^ = 0.0%). Combination interventions such as acupuncture plus rTMS also demonstrated high heterogeneity (*I*^2^ = 59.7%), while CCT plus tDCS was represented by a single study and therefore heterogeneity could not be estimated. The overall heterogeneity for MoCA was high (*I*^2^ = 82.5%). For the MBI, heterogeneity across intervention nodes was negligible, with *I*^2^ values of 0.0% for CCT and the overall analysis, indicating high consistency among included studies.

To further investigate the distribution of evidence and study-level heterogeneity, we generated forest plots and funnel plots for all comparisons (Supplementary Figure 11). These plots visualize the effect sizes (MDs) and 95% CrIs for each included trial across all intervention nodes. Consistent with our statistical analysis, the forest plots illustrate the high heterogeneity observed in the MoCA outcome (*I*^2^ = 82.5%). In contrast, the MBI displayed negligible heterogeneity (*I*^2^ = 0.0%), indicating highly consistent functional recovery outcomes across the included trials. Additionally, the comparison-adjusted funnel plots for both MoCA and MBI exhibited a balanced and symmetrical distribution of studies around the vertical zero line, indicating no significant evidence of publication bias or small-study effects in this NMA.

### Subgroup analyses based on intervention parameters

Subgroup analyses were conducted to investigate whether variability in tDCS treatment protocols contributed to heterogeneity in MoCA. As shown in Supplementary Figure 5a, studies using a 4 week treatment duration with 2 mA stimulation demonstrated a modest but consistent cognitive benefit (WMD = 1.08; 95% CI: 0.59–1.56; *I*^2^ = 0.0%), whereas a longer treatment duration of 8 weeks with 1 mA stimulation yielded a substantially greater effect size (WMD = 3.69; 95% CI: 2.98–4.40). When stratified by stimulation intensity alone (Supplementary Figure 5b), trials employing 5 Hz neuromodulatory protocols showed robust and highly consistent cognitive improvements (WMD = 4.26; 95% CI: 3.44–5.08; *I*^2^ = 0.0%). Importantly, heterogeneity was markedly reduced within most subgroups compared with the overall pooled estimate (overall *I*^2^ = 48.9%), indicating that differences in treatment duration and stimulation intensity were major contributors to between-study heterogeneity.

### Publication bias, evidence contribution, and sensitivity analyses

Potential publication bias was examined using comparison-adjusted funnel plots, which showed no substantial asymmetry, suggesting a low risk of small-study effects. Contribution plots indicated that interventions involving CCT, tDCS, and rTMS contributed the largest proportion of evidence within the network. Overall, the evidence distribution was sufficiently balanced, and the network structure was considered robust, supporting reliable indirect and direct comparisons (Supplementary Online Content).

Sensitivity analyses were conducted using a leave-one-out approach to evaluate the robustness of the pooled estimates. For cognitive outcomes assessed by the MoCA, sequential exclusion of individual studies did not materially alter the overall effect estimates, with all recalculated 95% CrIs overlapping those of the primary analysis (Supplementary Figure 6a). Similar findings were observed for functional outcomes measured by the MBI, with minimal variation in pooled estimates and no single study exerting a disproportionate influence on the results (Supplementary Figure 6b). These findings indicate that the results of the NMA are stable and not driven by individual studies, including those with higher risk of bias.

## Discussion

This study represents the first comprehensive NMA to systematically compare the efficacy of multiple non-pharmacological interventions for improving cognitive and functional outcomes in patients with PSCI. By synthesizing evidence from 23 RCTs involving 1,723 participants, we demonstrated that CCT combined with tDCS achieved the greatest improvement in cognitive performance as measured by the MoCA.

For functional recovery, as assessed by the MBI, tDCS and exercise-based rehabilitation emerged as the top-ranked interventions. These findings underscore the superior effectiveness of multimodal approaches compared to single-modality treatments, suggesting that synergistic effects may be achieved when combining neuromodulation with targeted behavioral interventions.

Importantly, the results support the principle of intervention-specific targeting—that is, matching treatment strategies to dominant impairment patterns. For patients with prominent cognitive deficits, CCT combined with tDCS may be the optimal choice, whereas those with motor-functional limitations may benefit more from tDCS alone or structured physical rehabilitation. This tailored approach may enhance the precision and efficiency of PSCI management in clinical practice.

### Comparison with previous studies and novel contributions

Several recent network meta-analyses have explored the comparative efficacy of non-pharmacological interventions for vascular dementia or PSCI, providing an important foundation for this field. However, the present study differs from these prior works in several key aspects and offers distinct, clinically relevant insights. First, compared with the large-scale NMA focusing on vascular dementia rather than PSCI (33), our study specifically targets PSCI as a distinct clinical entity, which differs from vascular dementia in terms of pathophysiology, disease trajectory, and rehabilitation potential. While that study included a wide range of interventions (e.g., fastigial nucleus stimulation and acupuncture-moxibustion combinations) and relied primarily on MMSE and ADL outcomes, our analysis emphasizes PSCI-specific populations, incorporates modern neuromodulation techniques and multimodal rehabilitation strategies, and prioritizes outcomes (MoCA and MBI) that are more sensitive to executive dysfunction and functional recovery commonly observed after stroke. Second, unlike the 2023 NMA that exclusively examined acupuncture-related therapies for PSCI (34), our study adopts a broader and more integrative intervention framework, simultaneously comparing cognitive training, non-invasive brain stimulation (tDCS and rTMS), exercise rehabilitation, acupuncture, and—critically—their combination therapies within a single network. This allows us to move beyond identifying the “best acupuncture modality” and instead address a more clinically relevant question: whether combining neurostimulation with cognitive rehabilitation yields superior outcomes compared with single-modality approaches. Our findings suggest that combination strategies, particularly CCT + tDCS, demonstrate the highest probability of cognitive benefit, highlighting the added value of multimodal intervention paradigms. Third, compared with the earlier 2022 Bayesian NMA that ranked acupuncture and TMS as the most effective therapies (35), our study incorporates more recent RCTs (up to 2025) and reflects the rapid evolution of cognitive rehabilitation technologies. Importantly, previous NMAs generally treated interventions as homogeneous nodes, whereas we explicitly addressed heterogeneity within intervention categories, conducted subgroup and sensitivity analyses, evaluated statistical heterogeneity (*I*^2^ values), assessed network inconsistency using node-splitting methods, and graded the overall certainty of evidence using GRADE. These methodological enhancements strengthen the robustness and interpretability of our conclusions.

Finally, our study provides new mechanistic and clinical insights by demonstrating that combination therapies consistently outperform single interventions in cognitive outcomes, supporting the hypothesis of synergistic effects between experience-dependent cognitive training and neuromodulation-induced plasticity enhancement. By integrating evidence quality assessment, heterogeneity exploration, and updated intervention comparisons, our work advances the existing literature from descriptive ranking toward evidence-informed clinical decision-making.

Taken together, while prior studies have laid important groundwork, the present NMA uniquely contributes by focusing on PSCI, incorporating up-to-date evidence, systematically evaluating combination therapies, and applying a more comprehensive methodological framework. These features allow our study to provide incremental yet meaningful advances in understanding the optimal non-pharmacological management of PSCI.

The superior efficacy of combination therapies, particularly the pairing of CCT with tDCS, can be attributed to synergistic neurobiological mechanisms that enhance cognitive rehabilitation outcomes. CCT primarily operates through task-specific and repetitive cognitive exercises, which engage specific cognitive networks and promote experience-dependent neural plasticity. This process facilitates functional reorganization in the brain, particularly in areas involved in working memory and other cognitive functions that are often impaired in post-stroke patients (36, 37). In contrast, tDCS acts as a neuromodulatory tool, enhancing cortical excitability by applying a weak, constant electrical current that shifts the resting membrane potential of neurons, thereby regulating neural activity and facilitating long-term potentiation (LTP)-like neuroplastic changes (38, 39). This modulation creates an optimal environment for learning, enabling more effective cognitive training. The combination of tDCS and CCT produces a state-dependent plasticity effect, where tDCS prepares the brain for cognitive training by enhancing its plasticity and excitability, thus amplifying the effects of the cognitive tasks (40). Furthermore, tDCS facilitates synaptic strengthening during cognitive training by modulating neurotransmitter systems like the NMDA receptors, which are crucial for learning and memory (41). This makes the brain not only more receptive to training but also helps consolidate the improvements gained from the exercises. Additionally, tDCS helps reorganize functional brain networks by targeting key regions like the dorsolateral prefrontal cortex (DLPFC), guiding cognitive networks toward more efficient patterns (14, 42). In clinical practice, it is recommended that patients with mild PSCI can benefit from standalone cognitive training or exercise rehabilitation, though combination therapies may provide further improvement, especially for cognitive functions that involve executive control. For patients with moderate PSCI, combining CCT with tDCS is likely to be more effective, as the enhanced neuroplasticity from neuromodulation provides greater support for the rehabilitation of more severely impaired cognitive functions. While the duration and frequency of treatment vary across studies, an intervention period of 4 to 8 weeks with 3–5 sessions per week appears to be effective for most patients. Future trials should explore specific dosing strategies tailored to the severity of cognitive impairment to optimize outcomes and inform personalized rehabilitation plans.

### Clinical implications and recommendations for practice

Given its superior performance in improving cognitive outcomes, we recommend that CCT combined with tDCS be considered a frontline early intervention, especially for patients with mild to moderate cognitive impairment following stroke. For patients presenting with prominent motor or functional limitations, tDCS alone or in combination with structured exercise rehabilitation appears to be the most effective approach. Our results also underscore the importance of interdisciplinary rehabilitation models, integrating neuromodulation, cognitive training, and traditional physical therapy into a cohesive intervention framework. Such a multimodal strategy may optimize treatment efficacy by targeting multiple recovery pathways simultaneously. In settings with limited resources, our findings support the use of high-ranking but lower-technology interventions—such as tDCS or basic CCT—as accessible, cost-effective options. These interventions require relatively modest infrastructure yet demonstrate clinically meaningful benefits, making them particularly suitable for broader implementation in under-resourced healthcare environments.

### Limitations and directions for future research

Several limitations of this NMA should be acknowledged. First, in terms of study distribution, most included trials were single-center studies with relatively small sample sizes. Notably, 21 of the 23 trials were conducted in China, with only one study each from Italy, Korea, and the United States. This pronounced geographical imbalance may restrict the applicability of findings to other ethnic populations, healthcare systems, and cultural contexts. Consequently, factors such as stroke subtypes, rehabilitation infrastructure, and varying delivery models across regions may influence the overall intervention effectiveness.

Second, regarding statistical robustness, a significant limitation is the high heterogeneity observed in cognitive outcomes, with an overall *I*^2^ of 82.5%. This variability likely arises from clinical heterogeneity in protocols, including differences in acupuncture points, stimulation intensities for tDCS/rTMS, and the specific content of cognitive training programs. As visualized in our forest plots, this high heterogeneity necessitated downgrading the certainty of evidence for MoCA to “Low” according to GRADE and CINeMA assessments.

Third, related to intervention consistency, there were substantial variations in protocols, including stimulation parameters (intensity and frequency), treatment duration, and session frequency. Such variability limits the comparability of effects across different interventions. While subgroup analyses and random-effects models were employed to partially address these variations, future studies must adopt standardized protocols to facilitate better reproducibility.

Fourth, from a methodological perspective, several included studies presented limitations in allocation concealment and blinding procedures. These issues were particularly common in trials involving acupuncture or physical interventions, where blinding of both participants and personnel is inherently challenging. Although sensitivity analyses excluding high-risk studies yielded results consistent with the primary findings, these methodological concerns may still lead to a potential overestimation of treatment effects.

Fifth, concerning the selection of outcome measures, we focused exclusively on the MoCA and MBI to ensure high sensitivity to executive dysfunction and functional recovery. Consequently, trials assessing cognition solely via the MMSE were excluded to avoid complex score transformation issues that could violate the assumptions of transitivity and consistency. However, the exclusion of MMSE-only studies may have reduced the comprehensiveness of the overall evidence base.

Finally, regarding the sustainability of effects, most trials assessed outcomes only immediately post-intervention, leaving the long-term durability of cognitive and functional improvements unclear. Future RCTs should incorporate follow-up periods of at least 6–12 months to better characterize the sustainability and clinical relevance of treatment effects over time.

## Conclusion

This NMA provides the first comparative efficacy ranking of non-pharmacological interventions for PSCI. CCT combined with tDCS demonstrated the greatest cognitive benefit, while tDCS and exercise rehabilitation were most effective for functional recovery. These findings support the adoption of tailored, multimodal strategies in clinical practice and highlight the need for high-quality, long-term trials to confirm durability and generalizability of treatment effects.

## Data Availability

The original contributions presented in the study are included in the article/Supplementary material, further inquiries can be directed to the corresponding author.
