# Supplementary material for: Comparative efficacy of non-pharmacological interventions for post-stroke cognitive impairment: a systematic review and network meta-analysis of randomized controlled trials
Source: Front Neurol. 2026 Mar 3;17:1644663. doi: 10.3389/fneur.2026.1644663 (PMC12991987; doi:10.3389/fneur.2026.1644663)
Supplement: Supplementary file 1 [file Supplementary_file_1.docx]

**Supplementary Methods**

**Search Strategy**

This systematic review and network meta-analysis employed a comprehensive search strategy, combining free-text terms and controlled vocabulary (e.g., MeSH, Emtree), across multiple databases, including PubMed, Embase, the Cochrane Library, Web of Science, and China National Knowledge Infrastructure (CNKI). The search covered all records from database inception through May 2025. The search terms encompassed post-stroke cognitive impairment, non-pharmacological interventions, cognitive training, neuromodulation, exercise rehabilitation, acupuncture, and randomized controlled trials. Additionally, the reference lists of included studies were manually screened to ensure thorough coverage and identify any potentially relevant studies.

**PubMed:**

("Post-Stroke Cognitive Impairment"[Mesh] OR "Stroke" AND "Cognitive Dysfunction"[tiab] OR "PSCI"[tiab] OR "Vascular Cognitive Impairment"[tiab])

AND

("Cognitive Training"[Mesh] OR "Computer-Based Cognitive Training"[tiab] OR "Cognitive Rehabilitation"[tiab] OR "Rehabilitation Exercises"[tiab] OR "Exercise Rehabilitation"[tiab] OR "Neuromodulation"[tiab] OR "Transcranial Direct Current Stimulation"[Mesh] OR "tDCS"[tiab] OR "Repetitive Transcranial Magnetic Stimulation"[Mesh] OR "rTMS"[tiab] OR "Acupuncture"[Mesh] OR "Electroacupuncture"[tiab] OR "Scalp Acupuncture"[tiab])

AND

("Randomized Controlled Trial"[Publication Type] OR "Randomised Controlled Trial"[tiab] OR "RCT"[tiab])

NOT

("Animals"[Mesh] NOT "Humans"[Mesh])

Filters: From 2000/01/01 to 2025/05/31; English

**Embase:**

('post-stroke cognitive impairment'/exp OR 'stroke cognitive dysfunction':ab,ti OR 'PSCI':ab,ti OR 'vascular cognitive impairment':ab,ti)

AND

('cognitive training'/exp OR 'computer-based cognitive training':ab,ti OR 'cognitive rehabilitation':ab,ti OR 'rehabilitation exercises':ab,ti OR 'exercise rehabilitation':ab,ti OR 'neuromodulation':ab,ti OR 'transcranial direct current stimulation'/exp OR 'tDCS':ab,ti OR 'repetitive transcranial magnetic stimulation'/exp OR 'rTMS':ab,ti OR 'acupuncture':ab,ti OR 'electroacupuncture':ab,ti OR 'scalp acupuncture':ab,ti)

AND

('randomized controlled trial'/exp OR 'randomised controlled trial':ab,ti OR 'RCT':ab,ti)

AND [english]/lim

AND [2000-2025]/py

**Web of Science Core Collection:**

TS=(("post-stroke cognitive impairment" OR "stroke cognitive dysfunction" OR "PSCI" OR "vascular cognitive impairment")

AND

("cognitive training" OR "computer-based cognitive training" OR "cognitive rehabilitation" OR "rehabilitation exercises" OR "exercise rehabilitation" OR "neuromodulation" OR "transcranial direct current stimulation" OR "tDCS" OR "repetitive transcranial magnetic stimulation" OR "rTMS" OR "acupuncture" OR "electroacupuncture" OR "scalp acupuncture")

AND

("randomized controlled trial" OR "randomized controlled trial" OR "RCT"))

Timespan: 2000-01-01 to 2025-05-31

Indexes: SCI-EXPANDED, SSCI, A&HCI, CPCI-S, CPCI-SSH, ESCI.

Language: English

**Cochrane Library (CENTRAL, Cochrane Reviews):**

("post-stroke cognitive impairment" OR "stroke cognitive dysfunction" OR "PSCI" OR "vascular cognitive impairment")

AND

("cognitive training" OR "computer-based cognitive training" OR "cognitive rehabilitation" OR "rehabilitation exercises" OR "exercise rehabilitation" OR "neuromodulation" OR "transcranial direct current stimulation" OR "tDCS" OR "repetitive transcranial magnetic stimulation" OR "rTMS" OR "acupuncture" OR "electroacupuncture" OR "scalp acupuncture")

AND

("randomized controlled trial" OR "randomized controlled trial" OR "RCT")

Publication Date from January 2000 to May 2025

**China National Knowledge Infrastructure (CNKI) Search Strategy:**

("Post-Stroke Cognitive Impairment" OR "Stroke Cognitive Dysfunction" OR "PSCI" OR "Vascular Cognitive Impairment")

AND

("Cognitive Training" OR "Computer-Based Cognitive Training" OR "Cognitive Rehabilitation" OR "Rehabilitation Exercises" OR "Exercise Rehabilitation" OR "Neuromodulation" OR "Transcranial Direct Current Stimulation" OR "tDCS" OR "Repetitive Transcranial Magnetic Stimulation" OR "rTMS" OR "Acupuncture" OR "Electroacupuncture" OR "Scalp Acupuncture")

AND

("Randomized Controlled Trial" OR "RCT")

Filters: From 2000/01/01 to 2025/05/31; Chinese and English
