# Supplementary material for: Comparative efficacy of non-pharmacological interventions for post-stroke cognitive impairment: a systematic review and network meta-analysis of randomized controlled trials
Source: Front Neurol. 2026 Mar 3;17:1644663. doi: 10.3389/fneur.2026.1644663 (PMC12991987; doi:10.3389/fneur.2026.1644663)
Supplement: Supplementary file 2 [file Supplementary_file_2.docx]

**Supplemental Online Content**

**Comparative Efficacy of Non-Pharmacological Interventions for Post-Stroke Cognitive Impairment: A Systematic Review and Network Meta-Analysis of Randomized Controlled Trials**

1. The risk of bias summary for studies included in the meta-analysis.
2. Rankogram.
3. Funnel plot.
4. Contribution plot.
5. Subgroup Analyses Based on Key Parameters.
6. Sensitivity analyses.
7. Heterogeneity (I^2^ values).
8. GRADE (Grading of Recommendations Assessment, Development and Evaluation).
9. Node-splitting approach.
10. CINeMA for risk assessment.
11. Forest plots and funnel plots.
12. **The risk of bias summary for studies included in the meta-analysis**

**a.**

**
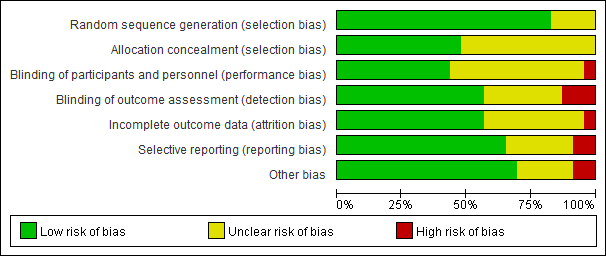
**

Risk of bias graph: review authors' judgements about each risk of bias item presented as percentages across all included studies.

**b.**


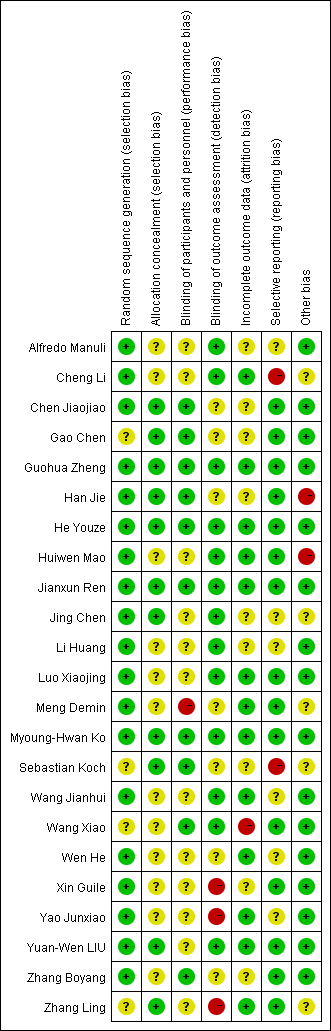


Risk of bias summary: review authors' judgements about each risk of bias item for each included study.

**2. Rankogram**

**a.**

**b.**

**(a) MoCA; (b) Modified Barthel Index;**

**3.** Funnel plot

**a.**

**b.**

**(a) MoCA; (b) Modified Barthel Index;**

**4.** Contribution plot.

**a.**

**b.**

**(a) MoCA; (b) Modified Barthel Index;**

**5.** Subgroup Analyses Based on Key Parameters

**a.**

**
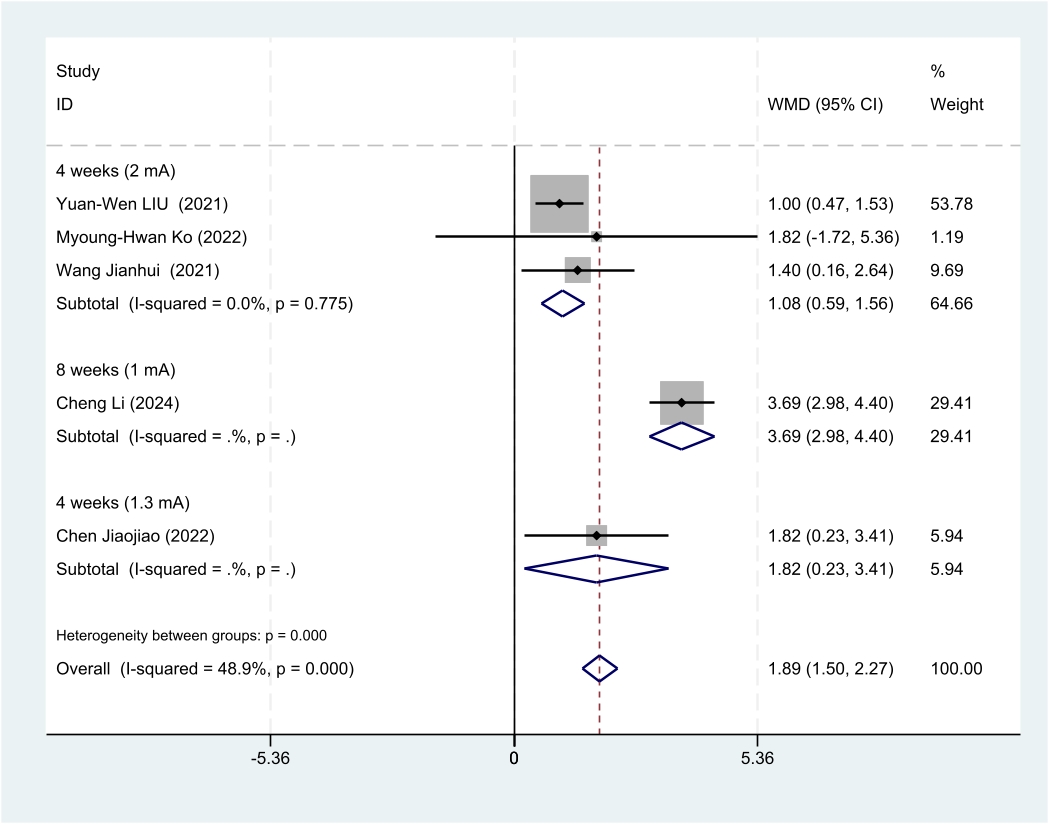
**

**b.**

**(a) MoCA: Subgroup Analysis Based on Treatment Duration and Stimulation Intensity of tDCS; (b) MoCA: Subgroup Analysis of tDCS: Impact of Treatment Duration and Stimulation Intensity**

6. Sensitivity analyses

**a.**

**b.**

**(a) MoCA; (b) Modified Barthel Index;**

7. Heterogeneity (*I^2^* values)

Supplementary Table 1. MoCA Summary of pairwise comparisons and statistical heterogeneity (I^2^) within intervention nodes.

| Comparison | No. of Studies | Sample Size, N | *I^2^* (%) | *P*-value | Heterogeneity Level |
| --- | --- | --- | --- | --- | --- |
| Acupuncture  Exercise rehabilitation  CCT  tDCS  CCT plus tDCS  Acupuncture plus rTMS  rTMS  Overall | 4  2  7  4  1  2  2  22 | 333  179  459  247  132  197  148  1695 | 85.8%  68.6%  70.3%  0.0%  -  59.7%  0.0%  82.5% | 0.000  0.074  0.003  0.733  -  0.115  0.953  0.000 | High  High  High  Negligible  N/A  High  Negligible  High |

Supplementary Table 2. Modified Barthel Index Summary of pairwise comparisons and statistical heterogeneity (*I^2^*) within intervention nodes.

| Comparison | No. of Studies | Sample Size, N | *I^2^* (%) | *P*-value | Heterogeneity Level |
| --- | --- | --- | --- | --- | --- |
| Exercise rehabilitation  CCT  tDCS  Overall | 1  4  1  6 | 48  234  80  362 | -  0.0%  -  0.0% | -  0.763  -  0.883 | N/A  Negligible  N/A  Negligible |

8. GRADE (Grading of Recommendations Assessment, Development and Evaluation)

| **Supplementary table 3. GRADE analysis: overall quality assessment and summary of findings** | | | | | |
| --- | --- | --- | --- | --- | --- |
| **Comparative Efficacy of Non-Pharmacological Interventions for Post-Stroke Cognitive Impairment: A Systematic Review and Network Meta-Analysis of Randomized Controlled Trials** | | | | | |
| **Patient or population**: Patients with post-stroke cognitive impairment (PSCI).  **Intervention**: Non-pharmacological interventions, specifically including computer-based cognitive training (CCT), transcranial direct current stimulation (tDCS), repetitive transcranial magnetic stimulation (rTMS), acupuncture, exercise rehabilitation, and their combinations.  **Comparison**: Control conditions (standard care or sham interventions as utilized in the included 23 randomized controlled trials). | | | | | |
| Outcomes | **Anticipated absolute effects^*^** (95% CI) | Relative effect (95% CI) | № of participants  (studies) | Certainty of the evidence (GRADE) | Comments |
| MoCA | **WMD 2.78** (2.25, 3.30) | - | 21 | ⨁⨁◯◯ LOW | Downgraded due to substantial heterogeneity (I^2^ = 82.5%) and risk of bias in some studies. Subgroup analysis indicates significant variation in effectiveness across different intervention modalities. |
| Modified Barthel Index | **WMD 7.02** (5.54, 8.50) | - | 6 | ⨁⨁⨁◯ MODERATE | Results are highly consistent (I^2^ = 0.0%). However, the evidence was downgraded due to the small number of included studies and potential implementation bias. |
| ***The risk in the intervention group** (and its 95% confidence interval) is based on the assumed risk in the comparison group and the **relative effect** of the intervention (and its 95% CI).  **CI:** Confidence interval; WMD: Weighted mean difference | | | | | |
| **GRADE Working Group grades of evidence** **High certainty:** We are very confident that the true effect lies close to that of the estimate of the effect **Moderate certainty:** We are moderately confident in the effect estimate: The true effect is likely to be close to the estimate of the effect, but there is a possibility that it is substantially different **Low certainty:** Our confidence in the effect estimate is limited: The true effect may be substantially different from the estimate of the effect **Very low certainty:** We have very little confidence in the effect estimate: The true effect is likely to be substantially different from the estimate of effect | | | | | |

9 Node-splitting approach

**a.**

**
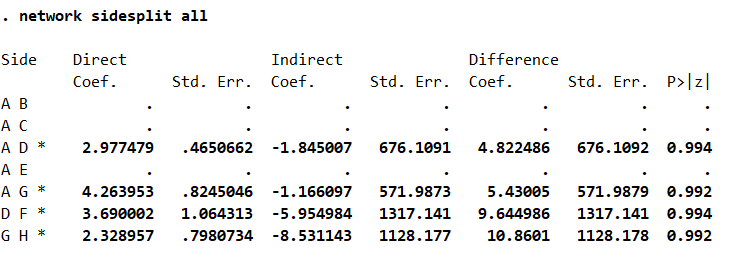
**

**b.**

**
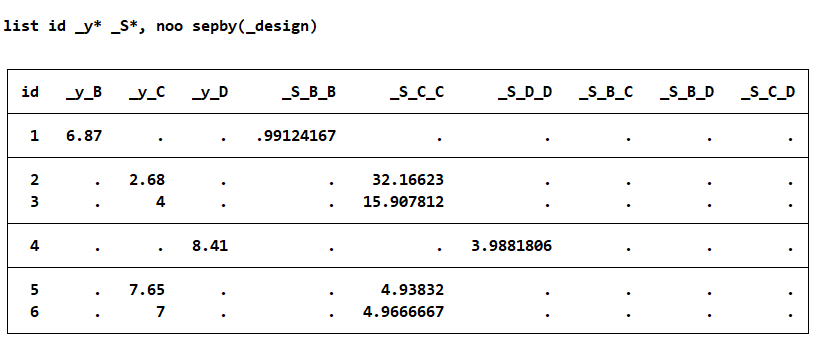
**

**(a) MoCA; (b) Modified Barthel Index;**

10. CINeMA for risk assessment

**
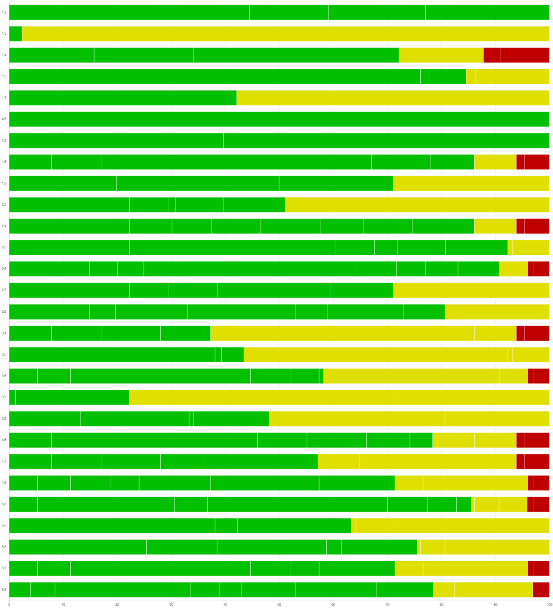
**

**
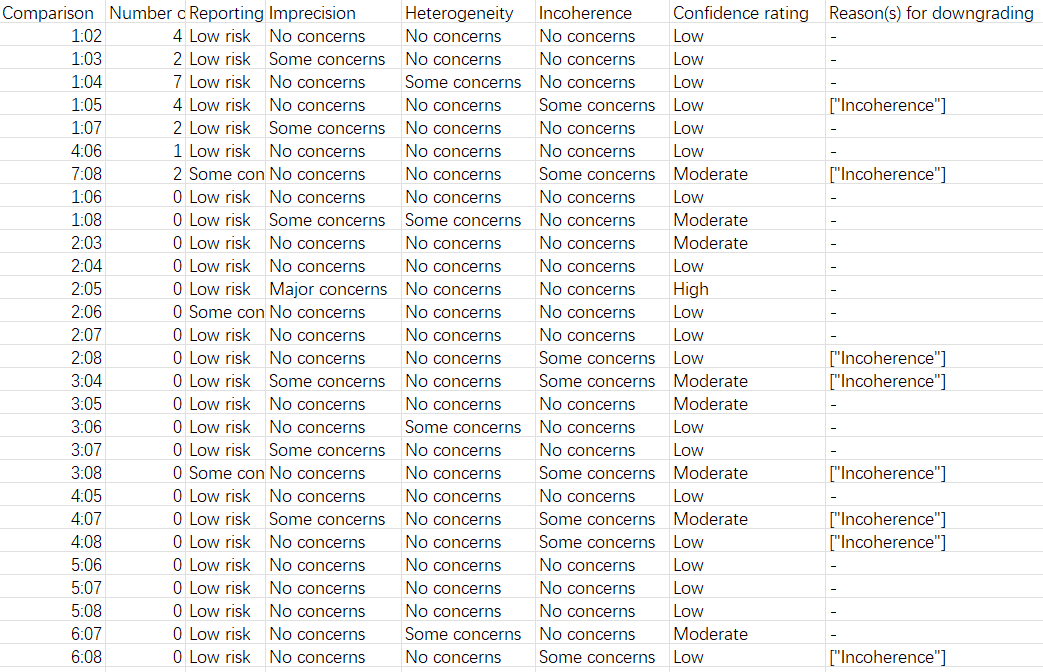
**

**11.** Forest plots and funnel plots

**a.**

**b.**

**c.**

**d.**

**(a)** Forest plots**-**MoCA; **(b)** Funnel plots**–**MoCA; **(c)** Forest plots-Modified Barthel Index; **(d)** Funnel plots-Modified Barthel Index;
